# Resource choice during ontogeny enhances both the short- and longer-term welfare of laying hen pullets

**DOI:** 10.1038/s41598-024-53039-7

**Published:** 2024-02-09

**Authors:** Regine Victoria Holt, Lena Skånberg, Linda J. Keeling, Inma Estevez, Ruth C. Newberry

**Affiliations:** 1 Department of Animal and Aquacultural Sciences, Faculty of Biosciences, Norwegian University of Life Sciences, Ås, Norway; 2 Department of Animal Environment and Health, Swedish University of Agricultural Sciences, Uppsala, Sweden; 3 Department of Animal Production, NEIKER, Basque Institute for Agricultural Research and Development, Arkaute, Spain; 4 IKERBASQUE, Basque Foundation for Science, Bilbao, Spain

**Keywords:** Animal behaviour, Developmental biology

## Abstract

We hypothesised that resource choice during early life contributes to both current and longer-term beneficial effects on animal welfare. We investigated this hypothesis in a longitudinal cross-over experiment with laying hen pullets (*Gallus gallus domesticus*) reared in pens with one or four litter and perch types, respectively (n = 8 pens/treatment, all providing ample and identical litter and perch space). After 4 weeks (chick period), half the pens were modified to provide the opposite treatment (juvenile period). After 11 more weeks, all groups were moved to novel, identical laying pens (adult period; Week 16–27). In support of our hypothesis, the opportunity to choose between multiple litter and perch variants was associated with higher levels of positively-valenced behaviours, including play as chicks and dustbathing as juveniles and adults, and lower levels of negatively-valenced behaviours, including feather pecking as chicks and juveniles and aggressive pecking as adults. Resource choice in the juvenile period also led to better juvenile and adult plumage condition, and greater growth as adults. We conclude that the opportunity to choose among different litter and perch types, instead of having only one type of each, had both short- and longer-term positive effects on the birds’ affective states and physical condition.

## Introduction

Domestic animals are typically provided with one form of each resource (e.g. a single type of litter or bedding, a single type of feed). When kept in large numbers, resources are distributed in a manner that emphasises habitat consistency and assumes animal uniformity. Decisions are made for animals to prevent them from making potentially costly choices (e.g. limiting movement that could risk injury). Homogeneous, monotonous environments leave animals with little freedom of choice, opportunities for decision-making or ability to develop a sense of agency through learning about the consequences of their choices^1,2^. They fail to accommodate variation in resource preferences over time and between individuals^3^ and limit opportunities to select different resource variants for different functions^4,5^. Further, they overlook the value of choice itself, which has been associated with activation of neural reward circuitry and affective liking^6^ and selected by animals over being assigned a preferred option without choice (reviewed by Refs.^7,8^).

Animal welfare has been conceptualised as varying along a continuum from poor to good, with negative experiences contributing to poor welfare and positive experiences to good welfare^9^. Thus, to improve the welfare of domestic animals, we should not only avoid animal suffering stemming from negative experiences but also promote positive affective states and long-term quality of life^10,11^. Providing an environment with multiple variants of different resources to choose from, referred to here as resource choice, could be an important source of environmental enrichment by promoting positive affective states and physical condition. This form of enrichment could be particularly relevant in animals with an evolutionary history of natural selection in complex, heterogeneous environments. Such natural environments are hypothesised to have favoured the evolution of affective and learning mechanisms that motivate decisions to engage in activities associated with fitness-promoting outcomes^12^ and optimise time and effort allocated to exploiting different resources^13^. In humans, opportunities for decision-making are reported to lead to a greater perception of freedom and control whereas lack of autonomous choice is perceived negatively, lowering motivation and sense of control^14^. Similarly, housing domestic animals with opportunities to exercise choice may give them an increased sense of control over their environment^15^, along with reward^16^ from using their selected options. Conversely, lack of opportunities to make choices may contribute to boredom^17^, as well as redirection of behaviour to inappropriate targets (e.g. Ref.^18^).

While some choice can be positive, having numerous choices is associated with psychological choice overload in humans^19,20^. Factors influencing human perceptions about choice include the degree to which options can be categorised according to usefulness, ease of evaluating trade-offs between choices, degree of time pressure to decide between available options, and the extent to which choices are perceived as final or reversible^20,21^. In animals, responses to options, and ultimately learning from choices, depends on the “price” of each option relative to immediate and future needs^22,23^, and memory of reward from previous engagement with the option^24,25^. These findings suggest that providing a moderate, but not overwhelming, level of resource choice may be optimal for domestic animal welfare. The resulting moderate level of challenge involved in decision-making may even contribute to flow, a state of engaged focus during which time seems to fly by, that hovers between states of boredom and anxiety and is juxtaposed against apathy^26,27^. Moreover, depending on the existing level of welfare, adding resource choice could lead to less poor welfare or further enhance already moderately good welfare. Because the level of challenge needed to stimulate flow and learning may increase over time due to increasing skill and “learning to learn”^28^, the level of resource choice having the most beneficial effects on animal welfare may increase with increasing experience.

The question then arises as to how early in ontogeny to provide resource choice. Exposure to a heterogeneous habitat in early life when habitat preference is imprinted^29^ may result in establishment of a broader habitat preference template. Developing familiarity with a broad constellation of environmental stimuli could aid adaptation to future environmental change because a greater proportion of the stimuli encountered following a change are likely to be at least somewhat familiar, speeding exploration of the novel components of a changed environment^24,30^. Furthermore, because neural plasticity is typically high at birth and declines through sensitive periods during ontogeny [reviewed by Ref.^31^], learning to navigate cognitive challenges imposed by resource choice may be more effective early during ontogeny, especially in precocial species, building confidence for future successful decision-making.

In animal husbandry, early exposure to resource choice would be of practical significance if it facilitates adaptation to subsequent planned environmental changes. For example, in pullets of the precocial domestic fowl (*Gallus gallus domesticus*) being raised for egg production, access to litter during the first few weeks after hatch has been associated with a reduced risk of feather pecking damage after transfer to the adult laying house^32,33^. Furthermore, exposure to perches starting before 4 weeks of age has been associated with a reduced risk of cloacal cannibalism as adults^34^. These findings suggest that the first four weeks of life may represent a sensitive period for initial exposure to litter and perches, with long-term consequences for their behaviour and health. Exposure to a choice of litter and perch types during the first four weeks may strengthen these effects by enhancing engagement with these resources, facilitating early development of skills in exploiting them for different functions.

Assessment of behavioural indicators of positive and negative affective states could provide insights into the extent to which pullet welfare is influenced by resource choice during different developmental periods. While affective states are coloured by how animals appraise their environment and experiences, they may be expressed in behaviour^35^. A positively-valenced state is inferred from play behaviour^36^, which usually occurs in young animals under safe, resource-abundant, fitness-promoting environmental conditions^37,38^ and may serve an adaptive function by generating opportunities for learning to cope with unexpected situations^39^. Play has been associated with activation of wanting and reward mechanisms in the brain^40^ and can generate location preference in a conditioned place preference paradigm^41^. It is most common in young individuals that have the most to learn, including domestic fowl (e.g. Ref.^42^), and has been correlated with growth^43^ and survival rate^44,45^ in some species. Dustbathing is another behaviour of domestic fowl inferred to be positively-valenced as its performance is responsive to opportunity situations^46^ and contributes to fitness by cleaning and fluffing up the feathers^47,48^. While both play and dustbathing are suppressed under unsafe environmental conditions, they rebound when conditions improve^42,49^, suggesting that they facilitate stress relief (e.g. Ref.^50^) and indicate a current, acute state of positive affect. If their prevalence is repeatedly found to be greater in one environment than another, based on spontaneous behaviour under undisturbed conditions, we interpret higher levels to indicate a higher level of positive experience in that environment at the flock level. Indicators of enhanced physical condition associated with these behaviours, such as higher body weight, and superior plumage and comb condition, would add to evidence of better welfare.

Behaviours associated with negatively-valenced affective states in domestic fowl could include vigilance, aggressive pecking, and feather pecking. Vigilance refers to alert behaviour expressed in response to potential danger (e.g. Refs.^51,52^ and may serve as an indicator of anxiety^53^. Aggressive pecking, which is directed towards the head and especially the comb, has been associated with frustration of access to resources, escalated resource defence, and physical deterioration due to wounds^54–56^. Severe feather pecking (i.e. pecks eliciting an avoidance response by the recipient) can impair thermoregulatory and flight ability and lead to cannibalism-related mortality^57,58^. Both aggressive and severe feather pecks can be painful for the receiver (e.g. Ref.^59^), and one instigator can harm multiple recipients. Accordingly, we infer that an elevated prevalence of these behaviours within a flock, and associated physical damage, indicates reduced welfare at the flock level.

Our aim was to investigate the hypothesis that welfare could be promoted in the short- and longer-term by rearing groups of laying hen pullets with resource choice in the form of four litter types and four perch types (Multi-choice) as opposed to a single type of each (Single-choice). Because we wished to investigate resource choice unconfounded by differences in the overall accessibility of litter and perches, we provided abundant and equal litter and perch space to all groups. Our goal was to achieve a good level of welfare in the Single-choice treatment, allowing for interpretation of any beneficial effects of the Multi-choice environment as signifying even better welfare. In this way, we sought to explore group-level welfare differences on the positive end of the welfare spectrum. Nevertheless, to gain a balanced view of the impact of our experimental treatments, we included both positively- and negatively-valenced welfare indicators in our assessment.

Building upon our previous research indicating some benefits of resource choice on adaptability and immune function in chicks^60,61^, we conducted a longer-term cross-over study comparing the behaviour of groups of pullets exposed to the Multi-choice *versus* Single-choice treatment during an early, chick rearing period (P1, Week 1–4), a later, juvenile rearing period (P2, Week 5–15), both rearing periods, or neither period (control). Spontaneous behavioural data were collected repeatedly by direct observation of undisturbed birds throughout rearing and during an adult period (P3, Week 16–27), after transfer to identical, complex adult laying pens containing novel litter and perch types, when any carry-over effects of the rearing treatments could be identified. Data on physical condition were collected at the end of each period. We predicted that, compared to the control environment, exposure to resource choice would increase frequencies of play and dustbathing behaviour (associated with positively-valenced affective states), whereas it would decrease frequencies of vigilance, aggressive pecking and severe feather pecking behaviour (associated with negatively-valenced affective states). We also expected it to result in improved physical condition, including higher growth rates and better feather and comb condition. We expected to find the greatest benefits of resource choice when provided throughout rearing (P1 and P2). Further, if it was important to add choice after gaining early experience, we expected that adding resource choice in P2 after the initial period without it would be more beneficial than removing it after providing it in P1. Contrarily, if early exposure to resource choice was important for “environmental imprinting” to a broad habitat template and establishing long-lasting positive habits and skills, we predicted that providing resource choice only in P1 would be more beneficial than providing it only in P2. Finally, we predicted that resource choice during rearing would have benefits that persisted into adulthood (P3), when all birds were kept under similar conditions.

## Results

**Figure 1:**
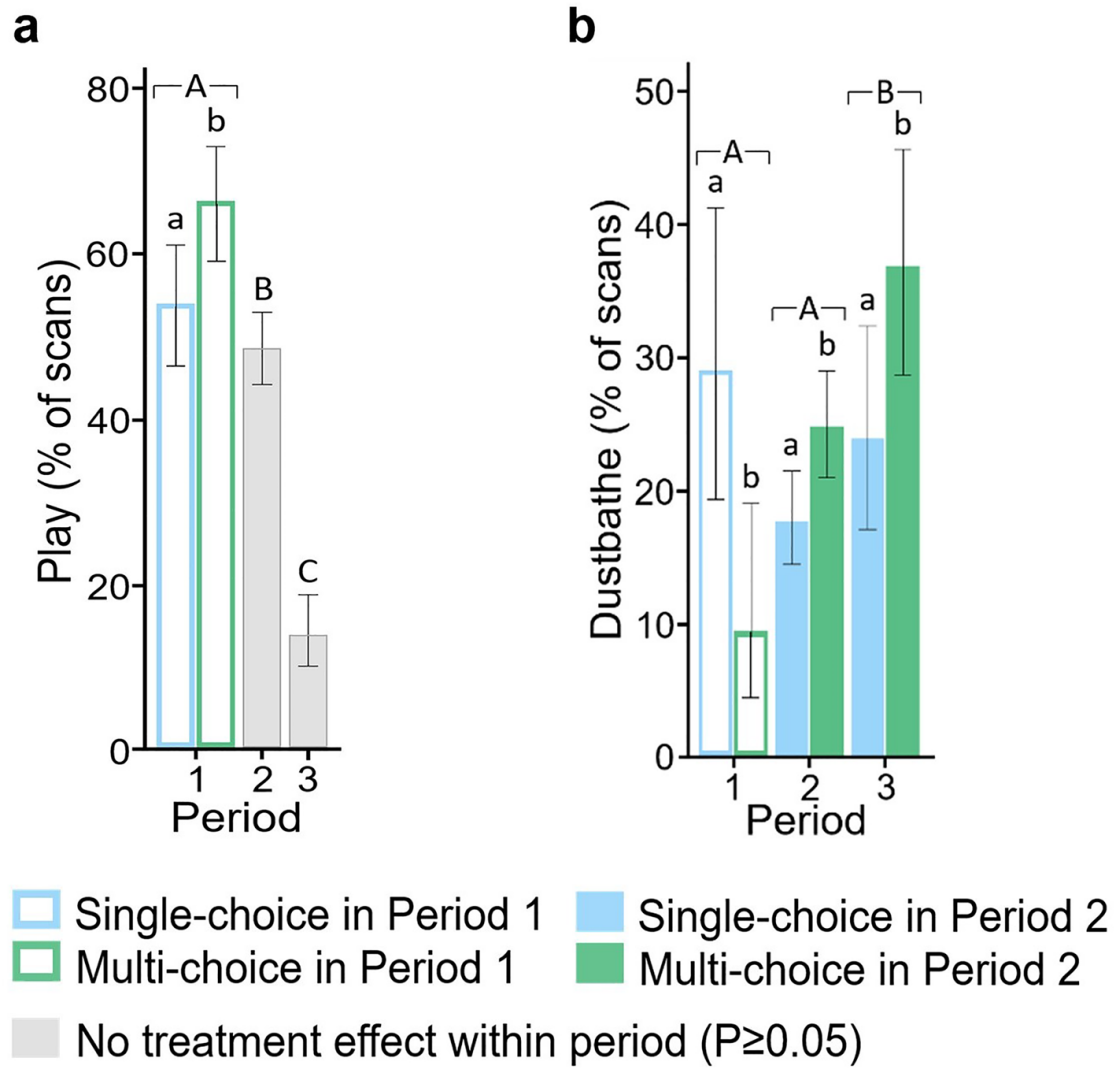
Resource choice affected positively-valenced behaviour (back-transformed mean % of scans with 95% CI) in different periods (1: Week 1–4; 2: Week 5–15; 3: Week 16–27). (**a**) play; (**b**) dustbathing. Bars with coloured outlines indicate an effect of the treatment experienced in Period 1 (Single-choice, blue; Multi-choice, green). Colour-filled bars indicate an effect of the treatment experienced in Period 2 (Single-choice, blue; Multi-choice, green). Uppercase letters indicate period differences and lowercase letters indicate treatment differences (P < 0.05).

Play in P1 was affected by the treatment in P1 (X^2^_1_ = 5.61, P = 0.018), occurring more often in the Multi-choice than in the Single-choice treatment (Fig. 1a). The occurrence of play declined across periods (χ^2^_2_ = 124.67, P < 0.001) and neither the P1 treatment nor the P2 treatment affected play during P2 or P3 (P > 0.05).

Dustbathing was observed more often in Single-choice than Multi-choice pens during P1 (χ^2^_1_ = 7.59, P = 0.006; Fig. 1b) but the P1 treatment did not affect dustbathing in P2 or P3 (P > 0.05). However, exposure to the Multi-choice *versus* Single-choice treatment in P2 resulted in a higher occurrence of dustbathing in both P2 (χ^2^_1_ = 6.80, P = 0.009) and P3 (χ^2^_1_ = 4.70, P = 0.030). Dustbathing also varied across periods (χ^2^_2_ = 11.13, P = 0.004), occurring more often in P3 than in P1 and P2.

**Figure 2:**
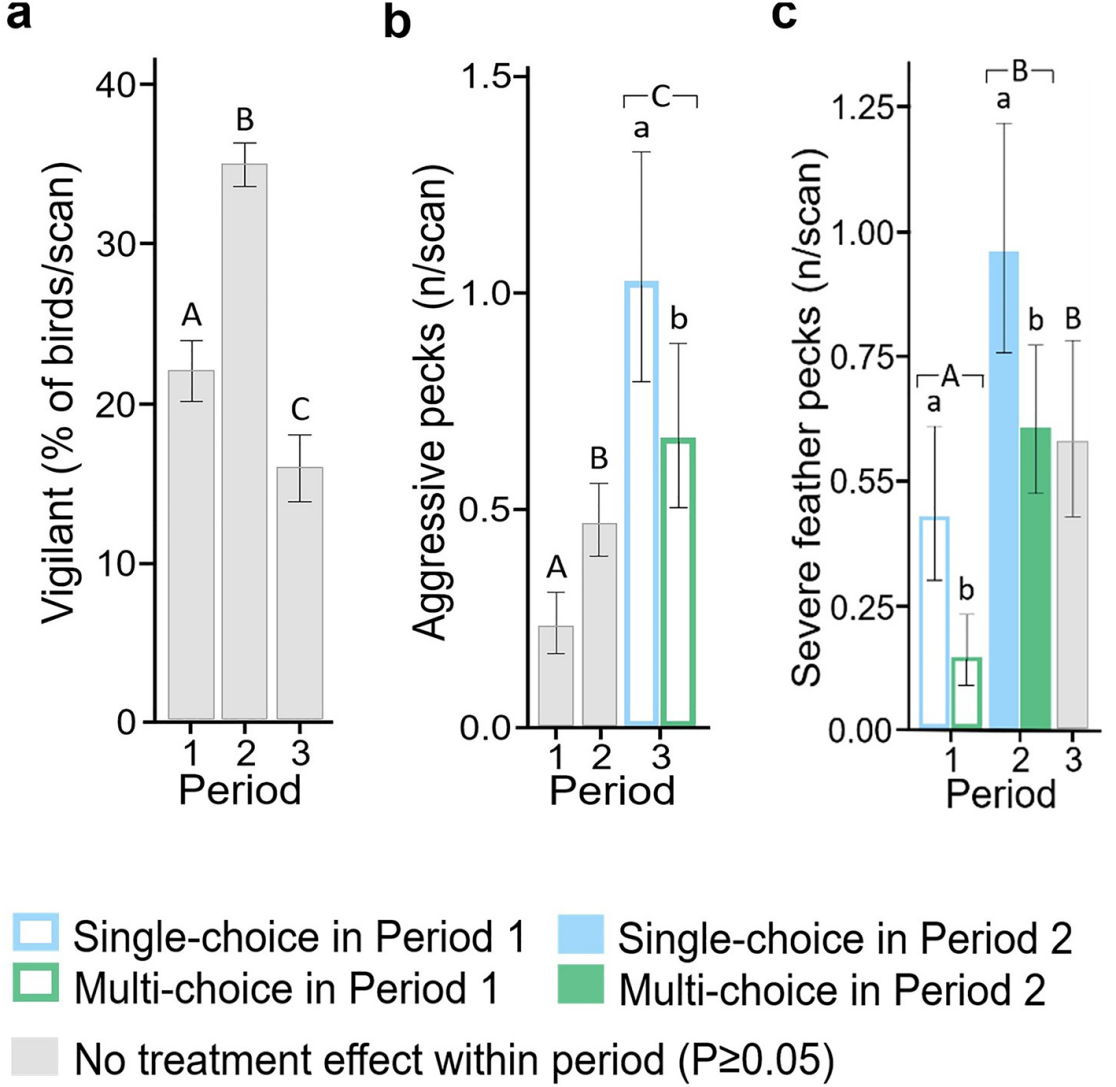
Resource choice affected negatively-valenced behaviour in different periods (1: Week 1–4; 2: Week 5–15; 3: Week 16–27). (**a**) vigilance (back-transformed least squares mean % of birds/scan), (**b**) aggressive pecking (back-transformed mean frequency/scan), and (**c**) severe feather pecking (back-transformed mean frequency/scan), with 95% CI. Bars with coloured outlines indicate an effect of treatment experienced in Period 1 (Single-choice, blue; Multi-choice, green). Colour-filled bars indicate an effect of treatment experienced in Period 2 (Single-choice, blue; Multi-choice, green). Uppercase letters indicate period differences and lowercase letters indicate treatment differences (P < 0.05).

The proportion of vigilant birds was not affected by current or previous treatment in any period (P > 0.05), but did differ between periods (F_2, 1694_ = 189.66, P < 0.001; Fig. 2a). The highest level of vigilance occurred in P2, followed by P1 and P3, respectively.

The frequencies of aggressive pecking in P1 and P2 were not affected by treatment in either P1 or P2 (P > 0.05). However, aggressive pecking in P3 was lower among hens that had been reared in the Multi-choice rather than the Single-choice treatment during P1 (χ^2^_1_ = 4.98, P = 0.026; Fig. 2b). The P2 treatment did not affect aggressive pecking in P3. Across periods, aggressive pecking increased as the pullets grew older (χ^2^_2_ = 50.26, P < 0.001).

The frequency of severe feather pecking was lower in the Multi-choice than in the Single-choice treatment both in in P1 (χ^2^_1_ = 12.93, P < 0.001) and P2 (χ^2^_1_ = 7.09, P = 0.008) (Fig. 2c). The treatment in P1 and P2 did not affect severe feather pecking during subsequent periods, respectively (P > 0.05). Across periods, pullets performed more severe feather pecking in P2 and P3 than in P1 (χ^2^_2_ = 43.36, P ≤ 0.001).

**Figure 3:**
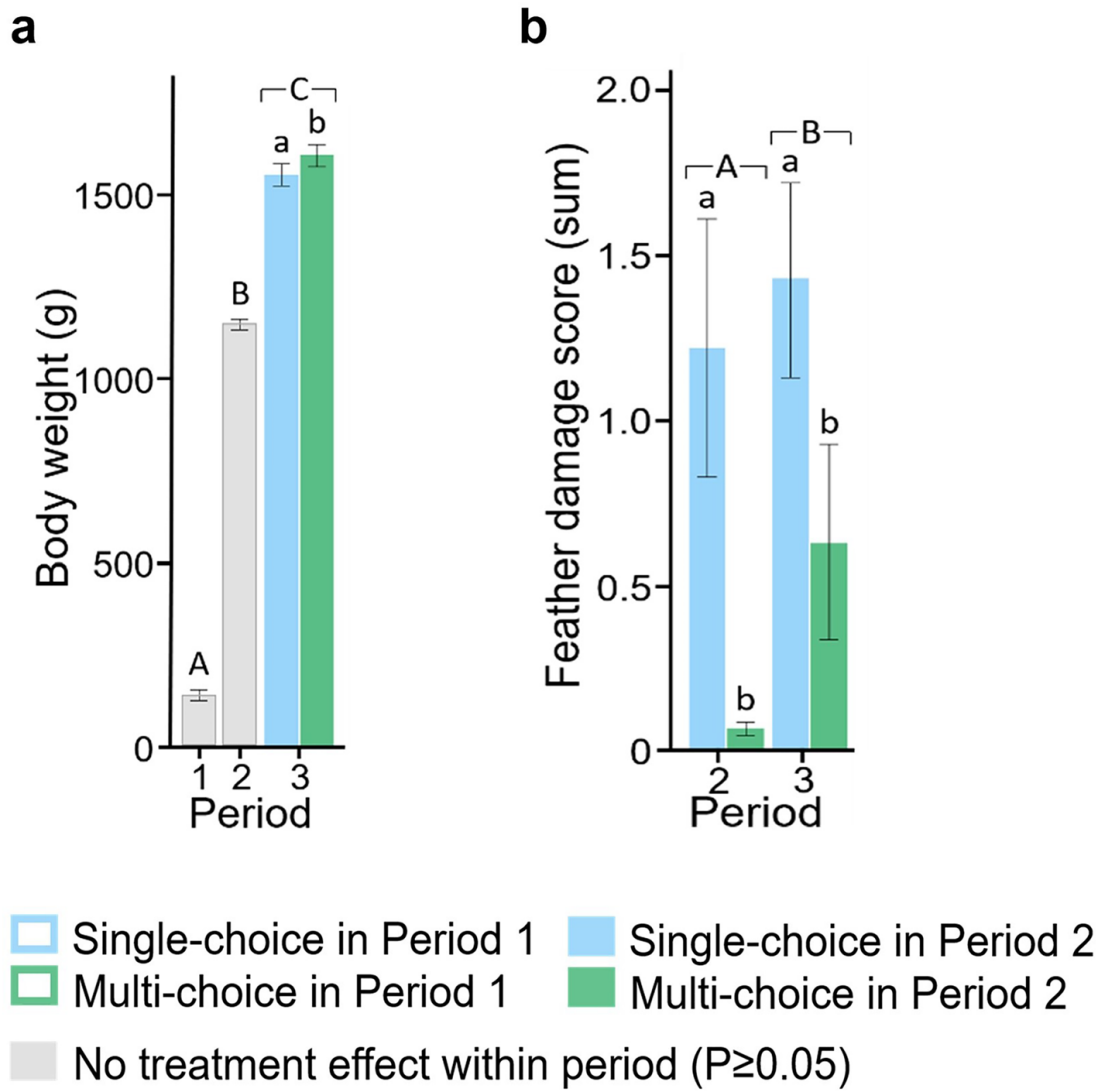
Resource choice affected physical condition in different periods (1: Week 1–4; 2: Week 5–15; 3: Week 16–27). (**a**) cumulative body weight (back-transformed least squares mean g), and (**b**) feather damage score (back-transformed mean of summed score), with 95% CI. A score of 0 = ≤ 50% of wing and tail feathers damaged, 1 = either wing or tail feathers ≥ 50–100% damaged and 2 = both wing and tail feathers ≥ 50–100% damaged. Colour-filled bars indicate an effect of treatment experienced in Period 2 (Single-choice, blue; Multi-choice, green). Uppercase letters indicate period differences and lowercase letters indicate treatment differences (P < 0.05).

From Week 1–27, five pullets died or were removed from the experiment (1.37% total mortality; P1: 0.55%; P2: 0.83%; P3: 0%), with mortality seemingly unrelated to treatment (too low for statistical comparison). No cases of cannibalism occurred (i.e. tissue loss due to injurious pecking). Body weights in P1 (Day 17) and P2 (Day 108–109) were unaffected by treatment during those periods (P > 0.05; Fig. 3a). However, pullets kept in the Multi-choice treatment in P2 had higher body weights and, thus, a higher growth rate, in P3 (Day 184–186) than those kept in the Single-choice treatment during P2 (F_1, 12.9_ = 7.21, P = 0.019).

Whereas the feather damage scores in P2 and P3 were not affected by the P1 treatment (P > 0.05), pullets kept in the Multi-choice treatment (*versus* the Single-choice treatment) in P2 had lower feather damage scores in both P2 (χ^2^_1_ = 8.77, P = 0.003) and P3 (χ^2^_1_ = 9.64, P = 0.002; Fig. 3b). We noted that the observed feather damage was mild and none of the birds had patches of broken or missing feathers. Comb wounds, which were recorded in P3, were unaffected by treatment in P1 or P2 (P > 0.05; arithmetic mean score with 95% CI on a scale from 1 to 3: 2.22 [2.14, 2.29]).

## Discussion

We hypothesised that early life exposure to a more heterogeneous environment with multiple litter and perch variants (resource choice) would have both short- and longer-term positive effects on the birds’ affective states and physical condition. In support of this hypothesis, we found that in different periods (though not consistently across all periods), pullets with the opportunity to choose between multiple litter and perch types displayed higher levels of positively-valenced behaviour (play and dustbathing), and lower levels of negatively-valenced behaviour (aggression and severe feather pecking). These results were accompanied by some indications of better physical condition related to rearing in the Multi-choice treatment (growth, feather condition). Furthermore, we detected treatment effects both during rearing when multiple choices were offered and in adulthood when all hens were housed in a similar environment.

As predicted, spontaneous play occurred more often in Multi-choice than Single-choice pens, though only as chicks (P1). The higher heterogeneity of the Multi-choice treatment presumably provided more learning opportunities compared to the less diverse Single-choice treatment in P1. Higher levels of play may have been stimulated and reinforced by reward associated with making choices and discoveries^6,7,39^. The lack of detected treatment differences in play later in rearing (P2) and as adults (P3) was likely related to a species-typical developmental decline in locomotor and social play during these periods, consistent with previous observations on chickens (e.g. Refs.^62,63^).

Also as predicted, the Multi-choice treatment resulted in more dustbathing as juveniles (P2), with carry-over into adulthood (P3), though unexpectedly less dustbathing was recorded in the Multi-choice than the Single-choice treatment in the chick period (P1). It is unlikely that the higher play in Multi-choice pens in P1 reduced opportunities for dustbathing because spontaneous play takes up only a small proportion of the behavioural time budget (e.g. Ref.^42^). Moreover, as we used 1–0 sampling at the group level, play and dustbathing could co-occur in the same scan. Although the heat lamp was centred over the litter trays and perches in P1, a slightly more preferred temperature in one litter tray than another may have encouraged dustbathing in that tray even if not containing an ideal litter material for dustbathing. For chicks in the Multi-choice treatment, the contrast in feedback from dustbathing in more *versus* less preferred dustbathing materials may have resulted in truncation of dustbathing bouts in less preferred materials^48^, resulting in less dustbathing overall than in the Single-choice pens. A localised microclimate effect within pens would have been less likely after removal of the heat lamps (P2), when pens were kept at room temperature. Furthermore, the Multi-choice birds may have been more adept at selecting to dustbathe in more optimal litter materials as juveniles following experience of dustbathing in the different litter choices as chicks, leading them to perform longer dustbathing sequences overall^48^. However, this explanation cannot account for higher dustbathing levels in the adults based on Multi-choice experience as juveniles, because all adults had the same type of litter (different from the types provided during rearing). Their Multi-choice experience as juveniles, including their lower level of feather pecking in that period, may have allowed them to function more harmoniously within their social group, enabling them to spend more time engaged in rewarding activities such as dustbathing^46^. Their higher adult growth rate is consistent with this explanation, suggestive of a predominance of parasympathetic as opposed to sympathetic neural activity. Overall, the number of scans in which dustbathing occurred was similar throughout rearing, consistent with previous observations across the rearing period^64^. Dustbathing increased in adulthood, possibly related to the greater feather mass of adults^65^, thereby requiring more dustbathing to keep the plumage in good condition.

We expected that lack of resource choice would lower perceived control over the environment, resulting in greater vigilance in Single-choice than Multi-choice pens. However, vigilance was not affected by choice provision in any period, maybe because the observations were conducted on undisturbed birds. While our measure of vigilance did not quantify intensity of the behaviour, it was our impression that the observed vigilance reflected routine monitoring of the surroundings rather than strong anxiety. We did find that vigilance varied with age, being highest in the juvenile period. An increase in vigilance from the chick to juvenile period is consistent with a reported increase in pullet vigilance while perching between 3 and 15 weeks of age^51^, possibly associated with vulnerability related to reduced flight ability during moulting of juvenile plumage. The drop in vigilance when adult could have been influenced by the greater boldness of adult hens^66,67^. The larger, more complex adult laying pens also provided higher elevated structures which may have enhanced the birds’ sense of security.

We detected no differences in the frequency of aggressive pecks between treatments until adulthood when, consistent with our prediction, hens that had experienced the Single-choice treatment as chicks performed more aggressive pecks. The lack of treatment differences in aggressive pecking during rearing support that the allocation of each resource variant in the Multi-choice treatment was sufficient and we did not inadvertently create competition for the most attractive options. The rising level of aggressive pecking with increasing age was probably related to the rise in reproductive hormones during puberty as described by McKeegan and Savory^68^, who also reported an increase in the frequency of aggressive pecking in laying hens after the onset of lay. The increase in aggressive pecking likely explains why we only detected a treatment difference in the adults. It is possible that the treatment difference was influenced by the higher level of play experience gained by Multi-choice pullets as chicks, which could have promoted the development of improved social skills and more affiliative social relationships among flock mates. We did not detect a corresponding treatment difference in comb wound scores in the adults, probably because individuals varied in opportunities for avoidance and, thus, the extent to which aggressive pecks inflicted skin damage. In small groups such as observed in the current study, a dominance hierarchy would have emerged in the juvenile period^69^, following which a bird’s position in the hierarchy would likely have had the greatest impact on severity of comb damage.

Severe feather pecking was less frequent in the Multi-choice than Single-choice treatment in the chick and juvenile rearing periods as predicted, although without carry-over to adults. The diverse litter types in the Multi-choice treatment could have been more effective in attracting and maintaining interest in foraging in the litter, reducing the risk of a shift in attention towards the feathers of flock mates. It appears that this was a direct effect of litter choice on current behaviour as, in adulthood when there was only one litter type (though multiple perch types), severe feather pecking was unaffected by rearing treatments. This result is also consistent with the observation that performance of severe feather pecking prior to the onset of lay is not a strong predictor of which hens will perform this behaviour in adulthood^70^. The higher level of juvenile feather pecking in the Single-choice than Multi-choice treatment was accompanied by higher feather damage scores that persisted into adulthood. However, although the recorded feather pecks were those eliciting immediate avoidance or vocal “complaints” by recipients, we did not find evidence that the feather pecking was leading to severe feather damage or cannibalism. Instead, the observed feather damage was mild, giving the plumage a somewhat dishevelled appearance. Therefore, the better juvenile and adult plumage condition of birds kept in the Multi-choice *versus* the Single-choice treatment as juveniles may have been more related to their higher levels dustbathing as juveniles and adults than their lower levels of juvenile feather pecking. Nonetheless, severe feather pecks occurring later in the juvenile period (after moulting of juvenile feathers) may account for some of the treatment difference in feather damage detected when adult.

Contrary to our predictions, we detected no interactions between the treatment experienced in the chick *versus* the juvenile period. Depending on the measured variable, access to resource choice was beneficial in one period or the other and when present in both periods, the effects were neither magnified nor antagonistic. Further, across the measured variables, we found no support for a more pronounced longer-term (P3) impact of resource choice in P1 as opposed to P2. These findings could be due to the differing age-related developmental trajectories of the metrics used in this study, with play being more prevalent when young, and dustbathing, aggression and feather pecking when older. Short-term positive incentive contrast (rebound) has been reported in chickens when opportunities for play and dustbathing have been boosted by giving access to more space or play objects^42^, and fresh litter^4^, respectively. Moreover, delayed access to litter and perches can have negative consequences by increasing the risk of feather pecking and cannibalism^32–34^, and the removal of enrichments has been reported to have negative outcomes on animal welfare in some contexts (e.g. Refs.^71–73^). However, our birds always had access to at least one type of litter and perch, and the litter was regularly refreshed, including at the time of the treatment switch in half the pens at the start of the juvenile period, which may have precluded lasting interaction effects. While simpler to provide resource choice for only a limited period during rearing, when viewed collectively across the measured welfare indicators, the beneficial effects detected in all periods support the continued provision of opportunities for resource choice throughout the whole rearing phase.

Our findings using multiple variants of litter and perches provide a basis for extending research to additional constellations of resource choice, and examination of the generalisability of the findings to other animal species. While we made observations at the group level, in future studies, it would be fruitful to investigate how different individuals within groups respond to multiple resource variants according to individual differences in personality and behavioural plasticity. Accommodating individual differences in resource selection could facilitate niche construction and the establishment of extended phenotypes^74^. Within large flocks of domestic poultry, this may increase opportunities for individuals to share resources equitably in time and space. For practical implementation, it will be necessary to establish optimal levels of accessibility to different resource variants under commercial conditions (e.g. area or quantity/animal, and frequency of replenishment if ephemeral) to avoid any adverse effects of resource competition. Promisingly, we did not find any indication that the levels of resource choice offered in the current study promoted aggressive or scramble competition as chicks or juveniles. To the contrary, birds in the Multi-choice rather than the Single-choice treatment as juveniles performed more dustbathing, less feather pecking and similar rates of aggression when juvenile, as well as having higher body weights as adults.

## Conclusions

While much research has been conducted on effects of adding occupational enrichments or structural complexity to reduce the negative effects of otherwise relatively barren environments, in this longitudinal cross-over study, we aimed to move the focus into the realm of positive animal welfare by adding a choice of litter and perch variants to an environment already enriched by the presence of litter and perches. Mechanisms underlying responses to resource choice could include stimulation of neural reward circuitry, learning to make fitness-promoting decisions about which substrate to use in different behavioural contexts, gaining experience that facilitates adaptation to environmental change, and enabling expression of individual preferences that deviate from the group average. While we do not know where our birds were on the continuum between poor and good welfare, our findings indicate that the opportunity to choose between several types of litters and perches during rearing (P1 and/or P2) was at times associated with higher levels of behaviour inferred to be positively-valenced and lower levels of behaviour inferred to be negatively-valenced, with some carry-over effects into adulthood (P3). Furthermore, these behavioural differences were associated with improvements in physical condition including better feather condition and increased growth. These results suggest that not only can early life in a multi-choice environment stimulate positive affective states in the short term, but it can also contribute to adaptive plasticity and longer-term quality of life. We conclude that resource choice offers a promising method for improving animal welfare in domestic animals.

## Methods

The study was conducted at the Swedish Livestock Research Centre of the Swedish University of Agricultural Sciences in Uppsala, SE. All methods were performed in accordance with the relevant guidelines and regulations. The procedures were approved by the Uppsala Animal Experiment Ethics Board Number 5.8.18-11549/2017, and complied with during the experiment, and all authors complied with the ARRIVE guidelines.

### Animal, housing, and management

Bovans Robust, white-feathered laying hen chicks (n = 364), with intact beaks and vaccinated against Marek’s disease, were obtained from a local commercial hatchery. The chicks were leg-banded and pseudo-randomly assigned to one of 16 groups in a manner that resulted in similar average bird weight and standard deviation across groups. Four groups comprised 22 chicks and the remaining groups comprised 23 chicks. The groups were housed separately from day 1 to 27 weeks of age, with twice daily routine care. The study was divided into three age periods.

### Chick rearing period (P1, week 1-4)

The rearing pens (240 × 120 × 180 cm), all located in one room, had heavy brown paper covering the walls to a height of 130 cm to block visual contact between pens. Each pen contained a drinker line with three nipples and a wire on top to discourage perching, and two feeders (23 cm Ø) placed on the floor. Litter (approximately 3 cm deep) was provided in four shallow plastic trays (71 × 35 × 3.5 cm), and on the concrete floor to avoid chilling of the chicks. Four perches (120 cm long) were placed above the litter trays with an initial height of 15 cm, and a hanging infrared heat lamp was centred over the litter trays and perches for localised brooding warmth (see *Experimental design* below for description of litter and perch types).

The litter trays were cleaned and refilled at 1- to 7-day intervals as needed to maintain continuous access to litter within. Coccidiosis vaccine was delivered in the drinking water in Week 2. At 3 weeks, the perches were raised to 45 cm, the water line was raised, and the ground feeders were replaced with a round hanging feed hopper (40 cm Ø). In Week 4, the leg bands were removed, and permanent wing tags were applied for individual identification. Starter feed and water were provided ad libitum. Room temperature, light schedule and ventilation were automatically controlled. Room temperature was kept at 25 °C. Lights (warm white light-emitting diode (LED)) were on for 20 h on Day 1, 18 h on Day 2, 16 h for the rest of the week, 14 h in Week 2, 13 h in Week 3 and 12 h in Week 4. Apart from a 15-min dawn and dusk at the start and end of each photoperiod, respectively, the mean light intensity in the pens was 18 lx at chick level (range: 7–37 lx).

### Juvenile rearing period (P2, Week 5-15)

At the beginning of Week 5, the heat lamp and floor litter were removed, the perches were raised to 55 cm, and the four shallow litter trays were exchanged for deeper trays (78 × 56 × 18 cm). The litter trays were cleaned and refilled once weekly to a depth of approximately 5 cm. The starter feed was exchanged for growing feed at 7 weeks of age. Room temperature was reduced to 20 °C at 8 weeks of age. The photoperiod was decreased by 1 h/week from 12 to 10 h and then held stable.

### Adult laying period (P3, week 16-27)

In Week 16, all groups were transferred to new, visually-isolated pens (362 × 356 × 297 cm) in another experimental room to simulate moving to a laying house. Each pen had a concrete-floored litter area (132 × 356 cm) containing crushed straw pellets as litter (finely-chopped 85% wheat straw, maximum length 1 cm, approximately 5 cm deep), and a raised, sloping (3.7°) plastic slatted area (230 × 356 cm, 29 cm high adjacent to the floor). Three shallow plastic platforms were attached to the wall above the floor (71 × 35 × 3.5 cm; 70, 155 and 220 cm high, respectively). Above the slatted area were a spruce wood perch (120 × 4.5 × 4.5 cm; 187 cm high), a slanted perch stand with four plastic perches (155 cm long; set at heights of 43, 96, 149 and 205 cm, respectively) and five low plastic perches (320 × 4 × 3 cm, 3 cm above the slats). Two hanging feed hoppers (40 cm Ø) containing layer feed, one bell drinker (40 cm Ø) and two metal colony nests (each 115 × 46 × 30 cm, with red vinyl door flap) were accessible from the slatted area. Room temperature was kept at 20 °C and the mean light intensity from warm white LED lights was 5.4 lx at hen level (range: 3–8 lx). The photoperiod, including a 15-min dawn and dusk, was 10 h to 20 weeks, then increased by 1 h weekly to 14 h. It was then kept steady to the end of the experiment (27 weeks), when the hens were adopted out to local hobby farmers.

### Experimental design

**Figure 4:**
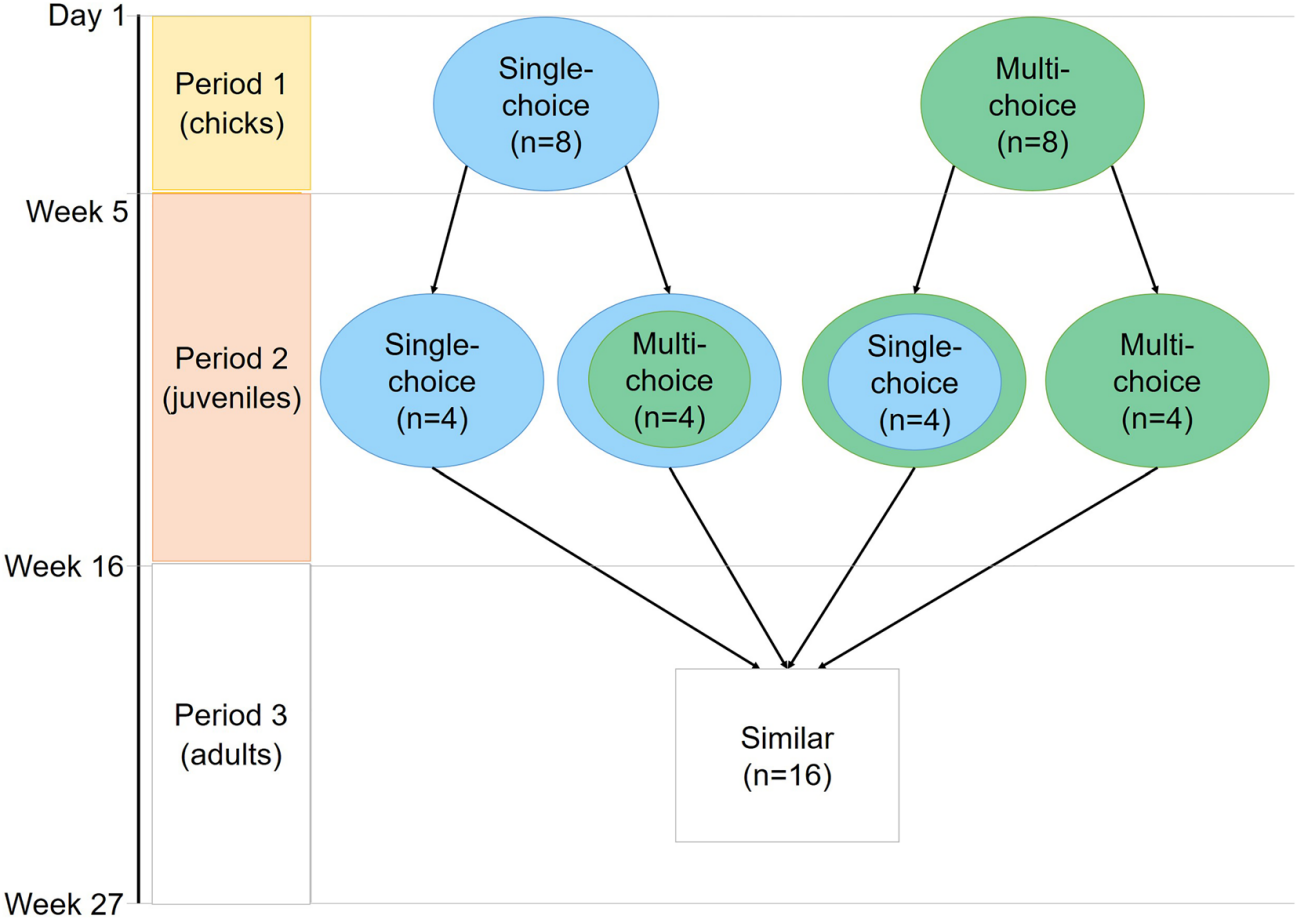
Effects of resource choice were investigated in a longitudinal cross-over experiment. In Period 1, groups (n = 16) of female laying hen chicks were assigned to one of two treatments: Single-choice with a single perch and litter type or Multi-choice with four perch and litter types. In Period 2, half the groups were switched to the opposite treatment. All groups were moved to similar pens for Period 3.

**Figure 5:**
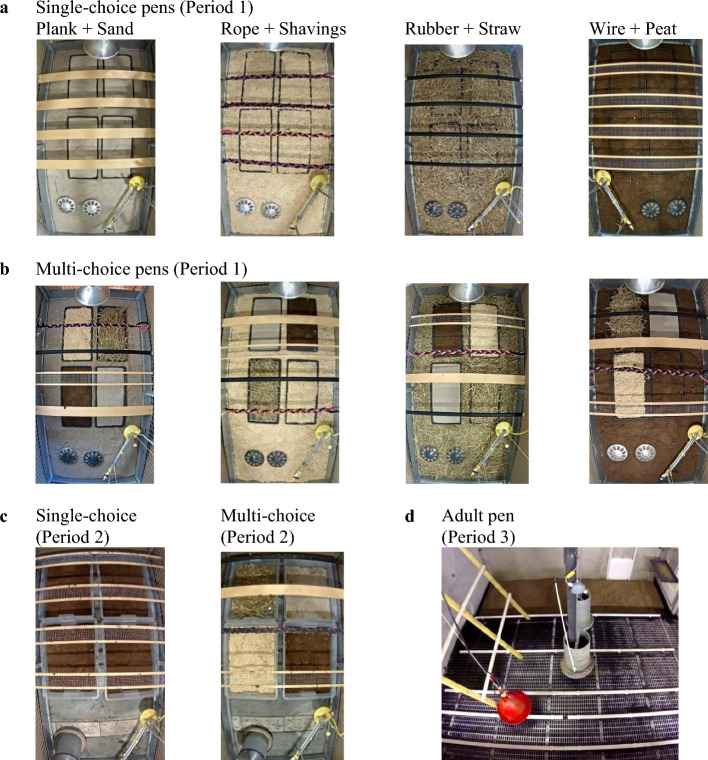
Resource choice was manipulated in pens with four litter trays and four perches. (**a**) Examples of Single-choice pens in Period 1 (Week 1–4), with a single litter and perch type. (**b)** Examples of Multi-choice pens in Period 1 having four litter and perch types. Type of floor litter, and the location of litter and perch types, were balanced across Multi-choice pens. (**c**) Example of Single-choice and a Multi-choice pen in Period 2 (Week 5–15). Perch and litter type combinations were as in Period 1, with litter in trays only. (**d**) An adult laying hen pen in Period 3 (Week 16–27; all adult pens were similar).

In P1, pens were assigned to one of two environmental treatments (Fig. 4). Single-choice pens (n = 8) had four perches of one perch type and four litter trays containing one litter type (with the same litter type on the floor). Based on our previous research^60,61^, one perch type was paired with one litter type such that preferred litter types were paired with less preferred perch types and vice versa, resulting in four combinations that were balanced across pens (Fig. 5a). This treatment focussed on the general effects of having only one perch and litter type, regardless of differences in specific litter and perch characteristics. Each Multi-choice pen (n = 8; Fig. 5b) contained all four perch types and all four litter types (one type/tray) used in the Single-choice treatment. The relative location of each perch and litter type within the pen was balanced across Multi-choice pens. In both treatments, the perch types were a pine plank (120 × 9.5 × 2 cm), a thick rope (three cotton horse leads braided together, 120 × 4 × 3 cm), a round rubber perch with wood core (120 cm long, 3.5 cm Ø) and a flat wire mesh perch in a spruce frame (120 × 13.5 × 1 cm, mesh openings 1 cm^2^). The litter types were fine-grained sand (maximum 0.3 mm Ø), wood shavings (dust-extracted mixed wood, mostly spruce), straw (100% wheat, long-cut) and peat (100% Sphagnum moss, > 0–3 cm particle Ø). One of these litter types also covered the pen floor (type balanced across pens). See Supplementary Table S1 for pen details.

At the beginning of P2, four pens/treatment were switched to the opposite treatment, resulting in four treatment combinations (Figs. 4; 5c): Single-choice in both periods, Single-choice to Multi-choice, Multi-choice to Single-choice and Multi-choice in both periods, arranged in a balanced block design with four blocks. The decision on the number of replicates was based on our previous work in which four replicate groups per treatment were sufficient to detect differences between Single-choice and Multi-choice treatments^60,61^. After the move to the laying room (P3), all groups were housed in similar pens (Fig. 5d) distributed across four blocks in a balanced block design. Blocking was done to control for possible small differences in environment across pens within experimental rooms.

### Data collection

Direct observations of the spontaneous behaviour of undisturbed birds in each pen were made by three experienced observers on one to three days each week from Week 3–23, with rounds of observations made across different times of day between 0900 and 1800 h, in a balanced order across pens. For each pen observation, the observer sat still in the aisle by the pen door for a 15-s familiarization period, then conducted an instantaneous scan of the number of vigilant pullets followed by a 3-min scan for the occurrence or number of events of the remaining behaviours in the ethogram (Table 1). To accommodate direct observations, we focussed on a limited number of easily detected, rapidly recognisable behaviour categories. These behaviours were also relatively uncommon, making it feasible to keep track of their occurrence during each scan. Because spontaneous performance of play and dustbathing is sporadic, we used 1–0 sampling to facilitate detection of treatment differences. Given that locomotion precedes social interaction during chicken play, making it difficult to differentiate between these forms of play, we combined locomotory and social play. A total of 368, 1056 and 288 observations per pen were made in P1, P2 and P3, respectively (Supplementary Table S2). We avoided collecting spontaneous behaviour data on days when birds were disturbed by researchers entering the pen for various purposes. Inter-observer concordance was monitored regularly and maintained at 90% or higher. In P1 and P2, the visual difference between treatments (Fig. 5) prevented blinding to the treatment under observation. Observers were blind to the previous treatments when collecting data during P3.

**Table 1:** Variables used to quantify pullet responses to resource choice comprised behaviour measures collected per pen and physical condition measures collected per individual.

Response variable	Description	Variable as analysed
Behaviour		
Play	One or more birds perform sudden spontaneous running (with twirls and/or wing-assisted movements, alone or in a group) and/or sparring (rapidly approaching a conspecific, often with wing movements, and standing staring with raised chest and upright neck; may include pecking towards, or jumping with feet towards, the other bird; recipient reciprocates or gives no visible reaction). Appears relaxed and non-harmful (without vocal “complaint”, injury or persistent unilateral withdrawal)	Occurrence (1/0) within pen during 3-min scan
Dustbathing	One or more birds perform vertical wing shaking, side lying and/or rubbing of head in litter while lying	Occurrence (1/0) within pen during 3-min scan
Vigilance	Bird sits or stands stationary on perch or floor, alert, with neck stretched and with either a fixed or rapidly scanning gaze	Proportion of birds vigilant within pen/instantaneous scan
Aggressive pecking	Bird delivers one or more pecks at head (comb to base of neck) of a conspecific with a stabbing or pulling movement, resulting in immediate reaction by receiver (moving away and/or vocalising sharply)	Number of pecks within pen/3-min scan
Severe feather pecking	Bird delivers one or more pecks at feathers or skin of a conspecific, at any body region excluding head (comb to base of neck), with stabbing or pulling movement, resulting in immediate reaction by receiver (moving away and/or vocalising)	Number of pecks within pen/3-min scan
Physical condition		
Mortality	Daily deaths (found dead and culled) registered by pen	Too rare for analysis
Body weight	Each bird weighed to nearest g on day 17 (Period 1), 108–109 (end of period 2) and 184–186 (end of period 3)	Body weight/bird (g)
Feather damage score	Proportion of split, frizzy and broken feathers, scored as 0 (< 50%) or 1 (≥ 50–100%) for each of two body regions/bird (wings, tail), on Day 108–109 (end of Period 2) and 184–186 (end of Period 3)	Summed score/bird
Comb wound score	Number of wounds on each bird’s comb, scored as 1 (0–3), 2 (4–6) or 3 (> 6), on Day 184–186 (end of period 3)	Score/bird

Physical condition data (Table 1) were obtained from 362 birds (Supplementary Table S3). The birds were weighed to the nearest g in P1 (Day 17), and at the end of P2 (Day 108–109) and P3 (Day 184–186). At the time each bird was weighed in P2 and P3, damage to the feathers of two body parts (wings, tail) was scored and summed for statistical analysis. Comb wounds were also scored during the final weighing in Week 27. Scores were determined by consensus between two scorers. Mortality was registered throughout the experiment.

### Statistical analysis

All statistical analyses were completed in R 4.3.1^75^, with two-tailed statistical significance set at P < 0.05. Due to repeated measures within pens, all variables were analysed using mixed models with pen as a random effect. Residual diagnostics plots for mixed models, computed using the DHARMa package^76^, were used to evaluate conformance with test assumptions and confirm model fit. Binary and count data were analysed with generalised linear mixed models (GLMM), whereby parameters were estimated based on maximum likelihood with Laplace approximation. A binomial distribution was applied to the 1/0 variables (playing, dustbathing). Overdispersion of the count variables (aggressive pecking, severe feather pecking) was addressed by specifying a negative binomial distribution. As the proportion of vigilant birds (vigilance) and cumulative body weight were continuous variables with Gaussian distribution, linear mixed models (LMM) were fit with restricted maximum likelihood (REML) and F-tests were conducted using the Kenward-Rogers approximation for degrees of freedom. The packages lme4^77^ and lmerTest^78^ were used for all GLMM and LMM analyses and the emmeans package^79^ was used for computing 95% confidence intervals (CI). For each response variable in P1, we evaluated the effects of block and P1 treatment and, in P2 and P3, we included block, P1 treatment, P2 treatment and their interaction. Observer was also included in behaviour models. Models were re-run excluding block and the interaction term as these factors were non-significant. Observer was also removed from the behaviour models as no consistent trends were observed. A separate model was used to assess changes in each response variable across periods (P1-P3). For presentation, proportions were transformed into percentages post-analysis (playing, dustbathing and vigilance).

Mortality was too low for statistical analysis. As feather damage and comb wound scores were ordinal variables with repeated sampling within pen, we used cumulative link mixed models (CLMM; ordinal package^80^ fit with Laplace approximation to evaluate the effects of block, P1 treatment, P2 treatment and their interaction on feather damage scores in P2 and P3, and comb wounds in P3. Block, and the interaction term, were non-significant and so dropped.

Type II Wald Chi-square tests (GLMM—car package^81^; CLMM – RVAideMemoire package^82^) and Type II F-tests (LMM – stat package^75^) were used to assess the significance of main effects. Planned pairwise comparisons of significant fixed effects were performed using the emmeans package^79^ before back-transformation of means to the response scale for presentation. For CLMM, this back-transformation required the mode “mean.class” to transform the ordinal levels of the response variable to a scale of 1–3 (due to three levels in ordinal response variables). Consequently, a constant of one (1.0) was subtracted from the pairwise comparison results of feather damage scores to transform the output to a scale of 0–2, a similar scale to the original summed scores. As comb wound scores already existed on a scale of 1–3, no post-pairwise comparison transformation was needed. See Supplementary Table S4 for back-transformed means with 95% CI for each model, Supplementary Table S5 for odds ratios from significant pairwise comparisons, and Supplementary Table S6 for back-transformed mean with 95% CI for each dependent variable by each treatment combination.

### Supplementary Information

Supplementary Tables.

## Data Availability

Pen-level data are provided in Supplementary Table S2, and individual-level data can be found in Supplementary Table S3.

## References

[1] Špinka M (2019). Animal agency, animal awareness and animal welfare. Anim. Welf..

[2] Špinka M, Wemelsfelder F, Appleby MC, Olsson IAS, Galindo F (2018). Environmental challenge and animal agency. Animal Welfare.

[3] Richter SH, Hintze S (2019). From the individual to the population–and back again? Emphasising the role of the individual in animal welfare science. Appl. Anim. Behav. Sci..

[4] Holt RV, Vas J, Vasdal G, Newberry RC (2023). A buffet of litters–Broiler chickens behave differently according to litter type and freshness. Appl. Anim. Behav. Sci..

[5] Skånberg L, Nielsen CBK, Keeling LJ (2021). Litter and perch type matter already from the start: Exploring preferences and perch balance in laying hen chicks. Poult. Sci..

[6] Leotti LA, Delgado MR (2011). The inherent reward of choice. Psychol. Sci..

[7] Englund MD, Cronin KA (2023). Choice, control, and animal welfare: Definitions and essential inquiries to advance animal welfare science. Front. Vet. Sci..

[8] Decker S, Lavery JM, Mason GJ (2023). Don’t use it? Don't lose it! Why active use is not required for stimuli, resources or “enrichments” to have welfare value. Zoo Biol..

[9] Broom DM (2023). Can positive welfare counterbalance negative and can net welfare be assessed?. Front. Anim. Sci..

[10] Boissy A (2007). Assessment of positive emotions in animals to improve their welfare. Physiol. Behav..

[11] Rault JL, Hintze S, Camerlink I, Yee JR (2020). Positive welfare and the like: Distinct views and a proposed framework. Front. Vet. Sci..

[12] Burgdorf J, Panksepp J (2006). The neurobiology of positive emotions. Neurosci. Biobehav. Rev..

[13] Fawcett TW (2014). The evolution of decision rules in complex environments. Trends Cogn. Sci..

[14] Moller AC, Ryan RM, Deci EL (2006). Self-determination theory and public policy: Improving the quality of consumer decisions without using coercion. J. Public Policy Mark..

[15] Wiepkema PR, Koolhaas JM (1993). Stress and animal welfare. Anim. Welf..

[16] Berridge KC, Robinson TE, Aldridge JW (2009). Dissecting components of reward: ‘Liking’, ‘wanting’, and learning. Curr. Opin. Pharmacol..

[17] Burn CC (2017). Bestial boredom: A biological perspective on animal boredom and suggestions for its scientific investigation. Anim. Behav..

[18] Rudkin C (2022). Feather pecking and foraging uncorrelated—The redirection hypothesis revisited. Br. Poult. Sci..

[19] Chernev A, Böckenholt U, Goodman J (2012). Choice overload: A conceptual review and meta-analysis. J. Consum. Psychol..

[20] Scheibehenne B, Greifeneder R, Todd PM (2010). Can there ever be too many options? A meta-analytic review of choice overload. J. Consum. Res..

[21] Gu Y, Botti S, Faro D (2013). Turning the page: The impact of choice closure on satisfaction. J. Consum. Res..

[22] Dawkins MS (1983). Battery hens name their price: Consumer demand theory and the measurement of ethological ‘needs’. Anim. Behav..

[23] Duncan IJH (1992). Measuring preferences and the strength of preferences. Poult. Sci..

[24] Vas J, BenSassi N, Vasdal G, Newberry RC (2020). Rewarding memories? Behaviour of broiler chickens towards peat in flocks with and without previous exposure to peat. Appl. Anim. Behav. Sci..

[25] Kacelnik A, Vasconcelos M, Monteiro T (2023). Testing cognitive models of decision-making: Selected studies with starlings. Anim. Cogn..

[26] Meehan CL, Mench JA (2007). The challenge of challenge: Can problem solving opportunities enhance animal welfare?. Appl. Anim. Behav. Sci..

[27] Hintze S, Yee JR (2023). Animals in flow—Towards the scientific study of intrinsic reward in animals. Biol. Rev. Camb. Philos. Soc..

[28] Harlow HF (1949). The formation of learning sets. Psychol. Rev..

[29] Stamps JA, Krishnan VV, Willits NH (2009). How different types of natal experience affect habitat preference. Am. Nat..

[30] Campderrich I (2019). Environmental complexity: A buffer against stress in the domestic chick. PLoS One.

[31] Tooley UA, Bassett DS, Mackey AP (2021). Environmental influences on the pace of brain development. Nat. Rev. Neurosci..

[32] Bestman M, Koene P, Wagenaar JP (2009). Influence of farm factors on the occurrence of feather pecking in organic reared hens and their predictability for feather pecking in the laying period. Appl. Anim. Behav. Sci..

[33] Tahamtani FM (2016). Effects of litter provision during early rearing and environmental enrichment during the production phase on feather pecking and feather damage in laying hens. Poult. Sci..

[34] Gunnarsson S, Keeling LJ, Svedberg J (1999). Effect of rearing factors on the prevalence of floor eggs, cloacal cannibalism and feather pecking in commercial flocks of loose housed laying hens. Br. Poult. Sci..

[35] Wemelsfelder F (2007). How animals communicate quality of life: The qualitative assessment of behaviour. Anim. Welf..

[36] Panksepp J (2011). The basic emotional circuits of mammalian brains: Do animals have affective lives?. Neurosci. Biobehav. Rev..

[37] Fraser D, Duncan IJH (1998). ‘Pleasures’, ‘pains’ and animal welfare: Toward a natural history of affect. Anim. Welf..

[38] Held SDE, Špinka M (2011). Animal play and animal welfare. Anim. Behav..

[39] Špinka M, Newberry RC, Bekoff M (2001). Mammalian play: Training for the unexpected. Q. Rev. Biol..

[40] Siviy SM, Panksepp J (2011). In search of the neurobiological substrates for social playfulness in mammalian brains. Neurosci. Biobehav. Rev..

[41] Trezza V, Damsteegt R, Vanderschuren LJ (2009). Conditioned place preference induced by social play behavior: Parametrics, extinction, reinstatement and disruption by methylphenidate. Eur. Neuropsychopharmacol..

[42] Liu Z, Torrey S, Newberry RC, Widowski T (2020). Play behaviour reduced by environmental enrichment in fast-growing broiler chickens. Appl. Anim. Behav. Sci..

[43] Brown SM, Klaffenböck M, Nevison IM, Lawrence AB (2015). Evidence for litter differences in play behaviour in pre-weaned pigs. Appl. Anim. Behav. Sci..

[44] Fagen R, Fagen J (2009). Play behaviour and multi-year juvenile survival in free-ranging brown bears, *Ursus*
*arctos*. Evol. Ecol. Res..

[45] Théoret-Gosselin R, Hamel S, Côté SD (2015). The role of maternal behavior and offspring development in the survival of mountain goat kids. Oecologia.

[46] Widowski TM, Duncan IJH (2000). Working for a dustbath: Are hens increasing pleasure rather than reducing suffering?. Appl. Anim. Behav. Sci..

[47] Olsson IAS, Keeling LJ (2005). Why in earth? Dustbathing behaviour in jungle and domestic fowl reviewed from a Tinbergian and animal welfare perspective. Appl. Anim. Behav. Sci..

[48] van Liere D (1992). The significance of fowls’ bathing in dust. Anim. Welf..

[49] Vestergaard KS, Damm BI, Abbott UK, Bildsøe M (1999). Regulation of dustbathing in feathered and featherless domestic chicks: The Lorenzian model revisited. Anim. Behav..

[50] Norscia I, Palagi E (2011). When play is a family business: Adult play, hierarchy, and possible stress reduction in common marmosets. Primates.

[51] Newberry RC, Estevez I, Keeling LJ (2001). Group size and perching behaviour in young domestic fowl. Appl. Anim. Behav. Sci..

[52] Zidar J, Løvlie H (2012). Scent of the enemy: Behavioural responses to predator faecal odour in the fowl. Anim. Behav..

[53] Campbell DLM (2019). An attention bias test to assess anxiety states in laying hens. PeerJ.

[54] Duncan IJH, Wood-Gush DGM (1971). Frustration and aggression in the domestic fowl. Anim. Behav..

[55] Estevez I, Newberry RC, Keeling LJ (2002). Dynamics of aggression in the domestic fowl. Appl. Anim. Behav. Sci..

[56] Leone EH, Estevez I (2008). Economic and welfare benefits of environmental enrichment for broiler breeders. Poult. Sci..

[57] McAdie TM, Keeling LJ (2000). Effect of manipulating feathers of laying hens on the incidence of feather pecking and cannibalism. Appl. Anim. Behav. Sci..

[58] Zhao Y, Xin H, Dong B (2013). Use of infrared thermography to assess laying-hen feather coverage. Poult. Sci..

[59] Gentle MJ, Hunter LN (1991). Physiological and behavioural responses associated with feather removal in *Gallus gallus var domesticus*. Res. Vet. Sci..

[60] Nazar FN, Skånberg L, McCrea K, Keeling LJ (2022). Increasing environmental complexity by providing different types of litter and perches during early rearing boosts coping abilities in domestic fowl chicks. Animals.

[61] Skånberg L, Newberry RC, Estevez I, Kelling LJ (2023). Environmental change or choice during early rearing improves behavioural adaptability in laying hen chicks. Sci. Rep..

[62] Lundén G (2022). Play ontogeny in young chickens is affected by domestication and early stress. Sci. Rep..

[63] Rayner AC, Newberry RC, Vas J, Mullan S (2020). Slow-growing broilers are healthier and express more behavioural indicators of positive welfare. Sci. Rep..

[64] Keeling LJ, Newberry RC, Estevez I (2017). Flock size during rearing affects pullet behavioural synchrony and spatial clustering. Appl. Anim. Behav. Sci..

[65] Xie WY (2020). Comparison of nonlinear models to describe the feather growth and development curve in yellow-feathered chickens. Animal.

[66] Hocking PM, Channing CE, Waddington D, Jones RB (2001). Age-related changes in fear, sociality and pecking behaviours in two strains of laying hen. Br. Poult. Sci..

[67] Albentosa MJ, Kjaer JB, Nicol CJ (2003). Strain and age differences in behaviour, fear response and pecking tendency in laying hens. Br. Poult. Sci..

[68] McKeegan DEF, Savory CJ (1998). Behavioural and hormonal changes associated with sexual maturity in layer pullets. Br. Poult. Sci..

[69] Rushen J (1982). The peck orders of chickens: How do they develop and why are they linear?. Anim. Behav..

[70] Newberry RC, Keeling LJ, Estevez I, Bilčík B (2007). Behaviour when young as a predictor of severe feather pecking in adult laying hens: the redirected foraging hypothesis revisited. Appl. Anim. Behav. Sci..

[71] Bateson M, Matheson SM (2007). Performance on a categorisation task suggests that removal of environmental enrichment induces ‘pessimism’ in captive European starlings (*Sturnus vulgaris*). Anim. Welf..

[72] Morano R, Hoskins O, Smith BL, Herman JP (2019). Loss of environmental enrichment elicits behavioral and physiological dysregulation in female rats. Front. Behav. Neurosci..

[73] Smith BL (2017). Behavioral and physiological consequences of enrichment loss in rats. Psychoneuroendocrinology.

[74] Edelaar P, Otsuka J, Luque VJ (2023). A generalised approach to the study and understanding of adaptive evolution. Biol. Rev..

[75] R Core Team. R: A language and environment for statistical computing. *R Foundation for Statistical Computing, *https://www.r-project.org/ (2023).

[76] Hartig, F. DHARMa: residual diagnostics for hierarchical (multi-level/mixed) regression models. R package version 0.4.6. https://cran.r-project.org/web/packages/DHARMa (2022).

[77] Bates D, Mächler M, Bolker BM, Walker SC (2015). Fitting linear mixed-effects models using lme4. J. Stat. Softw..

[78] Kuznetsova A, Brockhoff PB, Christensen RHB (2017). lmerTest package: Tests in linear mixed effects models. J. Stat. Softw..

[79] Lenth, R. et al*.* emmeans: Estimated marginal means, aka least-squares eans. Version 1.8.8. https://cran.r-project.org/web/packages/emmeans (2023).

[80] Christensen, R. H. B. ordinal - regression models for ordinal data. Version 2022.11-16. https://cran.r-project.org/web/packages/ordinal (2022).

[81] Fox J, Weisberg S (2019). An R Companion To Applied Regression.

[82] Hervé, M. RVAideMemoire: Testing and plotting procedures for biostatistics. Version 0.9-83-2. https://cran.r-project.org/web/packages/RVAideMemoire (2023).

